# The peculiarity of the metal-ceramic interface

**DOI:** 10.1038/srep11460

**Published:** 2015-06-19

**Authors:** Zaoli Zhang, Yao Long, S. Cazottes, R. Daniel, C. Mitterer, G. Dehm

**Affiliations:** 1 Erich Schmid Institute of Materials Science, Austrian Academy of Sciences, Jahnstraße 12, Leoben A-8700, Austria; 2 Laboratory of Computational Physics, Institute of Applied Physics and Computational Mathematics, P.O. Box 8009, Beijing 100088, China; 3 Department Physical Metallurgy and Materials Testing, University of Leoben, Franz-Josef-Strasse 18, Leoben A-8700, Austria; 4 Max-Planck-Institut für Eisenforschung, D-40237 Düsseldorf, Germany

## Abstract

Important properties of materials are strongly influenced or controlled by the presence of solid interfaces, i.e. from the atomic arrangement in a region which is a few atomic spacing wide. Using the quantitative analysis of atom column positions enabled by C_S_-corrected transmission electron microscopy and theoretical calculations, atom behaviors at and adjacent to the interface was carefully explored. A *regular variation* of Cu interplanar spacing at a representative metal-ceramic interface was experimentally revealed, i.e. Cu-MgO (001). We also found the periodic fluctuations of the Cu and Mg atomic positions triggered by the interfacial geometrical misfit dislocations, which are partially verified by theoretical calculations using empirical potential approach. Direct measurements of the bond length of Cu-O at the coherent regions of the interface showed close correspondence with theoretical results. By successively imaging of geometrical misfit dislocations at different crystallographic directions, the strain fields around the interfacial geometrical misfit dislocation are quantitatively demonstrated at a nearly three-dimensional view. A quantitative evaluation between the measured and calculated strain fields using simplified model around the geometrical misfit dislocation is shown.

The electrical properties in electronic industry are controlled by various interfaces, such as metal-ceramic and metal-semiconductor interfaces. In the fields of semiconductor technology and surface engineering, the metal-oxide interfaces are encountered frequently and playing a decisive role as they control the properties of metal–ceramic composites, protective coatings and thin metal–ceramic films in electronic devices etc. Although this obvious practical importance, our basic understanding of interfaces is still in its infancy, and lacks a fundamental correlation of interface structure and materials properties. The importance of interfaces essentially lies in the fact that physical and chemical properties may change dramatically at or near the interface itself. The significance of metal-ceramic interfaces in so many technological relevant composite materials and thin film electronic devices is strongly reflected in the continuing and extensive studies for many decades^1^,^2^,^3^,^4^,^5^,^6^,^7^,^8^,^9^,^10^,^11^. It is apparent that atoms at or near the interface do not all possess the same local environment. Thus, characterizing the local atomic structure at or adjacent to the interface becomes of vital importance as it controls the resulting properties. Although massive investigations of dislocation core structure of interfacial misfit dislocations have been performed using high–resolution transmission electron microscopy (HRTEM)^3^,^7^, the accurate and quantitative description on the local atomic structure is so far less advanced due to the limitations of lens aberration of the microscopes. This is especially true for semi-coherent interfaces, i.e. metal-oxide interfaces. The unambiguous knowledge of the exact atomic configurations and behaviors within a few layers at or adjacent to the interface is still scarce. This limitation can now be overcome with spherical aberration (C_S_)-corrected HRTEM. Now an abundance of knowledge on atomic scale structure from very local position can be gained. The intrinsic physics of materials is possible to be directly read out of the HRTEM images^12^,^13^. This largely prompts a new view on interface controlled materials, such as metal-ceramic composites and their interface structures. It is reasonably anticipated that a novel understanding can be achieved, and the physics behind metal-ceramic interfaces can be atomically unveiled when using the picometer-scale precision C_S_-corrected HRTEM^12^, and combined with the theoretical calculations.

Due to the mismatch of the two lattices, a dislocation network exists at metal-ceramic interfaces. To discriminate between subtle differences in the interfacial structure it would be beneficial to observe the interfaces edge-on along two different directions at the atomic scale, which is hardly possible in normal transmission electron microscopy (TEM). However, the image-side C_S_-corrected microscopes with their wide *pole-piece gap* enabling large tilt angles and an ultrahigh-resolution that are fortunately available in our microscope provide a possibility to study the interface in two projections by successively atom-resolved imaging in e.g. [100] and [110] zone axes. This will help achieving a better understanding of misfit dislocation structures and local strain variations at a nearly three-dimensional view^14^.

The Cu-MgO interface is a model system for analyzing metal-ceramic interfaces^1^,^2^,^3^,^4^,^5^,^6^,^7^,^8^,^9^,^10^,^11^. Both Cu and MgO are *fcc* lattices, and due to their different lattice parameter (*a*_MgO_ = 0.42105 nm and *a*_Cu_ = 0.36148 nm), a large mismatch of 14.1% exists between the two lattices. The ratio of the metal and oxide lattice constants can be approximated by a simple ratio: 7*a*_Cu_ ≈ 6*a*_MgO_ for Cu-MgO (001). For epitaxial Cu films grown on (001) MgO substrate, the dislocation network was found to lie along 〈100〉 directions with a Burgers vector of ½*a*_Cu_ 〈100〉 deduced from HRTEM images in an earlier study^15^, which is in agreement with other report^4^. On the other hand, as the interface possesses a large misfit, it could not be semi-coherent anymore, and the interface should be categorized into incoherent, forming a geometrical misfit dislocation (GMD) network. In this work, combining quantitative atom position measurements with theoretical calculations, and successively imaging of the same interface position along two different crystallographic orientations, we present a picometer-scale understanding of atom behaviors at and adjacent to the Cu-MgO interface.

## Experimental sections

The Cu-MgO interface is a model system for metal-ceramic interfaces. Both Cu and MgO are *fcc* lattices, and a large mismatch (14%) exists. The ratio of the metal and oxide lattice constants can be approximated by a simple ratio: 7*a*_Cu_ ≈ 6*a*_MgO_ for Cu-MgO (001). The epitaxial Cu film was deposited by using an unbalanced direct current magnetron sputtering system at a substrate temperature of 350 °C. Prior to deposition, a polished MgO (001) substrate was cleaned with successive rinses in ultrasonic baths of trichloroethylene, acetone, isopropyl alcohol, and deionized water and thermally degassed at 800 °C in vacuum. The 2 μm thick Cu layers were then sputtered in Argon atmosphere at 1 Pa total pressure, using a 99.99% pure Cu target, a power of 2 kW and at floating potential. Further details are reported in^15^. Possible point defects that form in the sputter-deposited Cu layer are expected to anneal out within seconds by Cu self-annealing at 350 °C^16^.

The TEM sample was specially cut at an angle of 22.5° between [001] and [011] orientations (supplementary, Fig. S1), enabling to reach both zone axes by tilting in the microscope for imaging the identical interface region. The TEM specimen was prepared by wedge Tripod polishing followed by a final ion milling at energy of 4.0 eV for a few minutes.

A 200 kV field emission TEM/STEM (JEOL 2100F) equipped with an image-side C_S_-corrector and particularly, with a large pole-piece gap in objective-lens that enables a tilting range of ±30–35° in combination with the high-resolution pole-piece lens employed, was used in this study. The microscope demonstrates a resolution of 1.2 Å at 200 kV. The alignment of the C_S_-corrector was performed using the CEOS software based on the aberration measurements deduced from Zemlin tableaus. Eventually, the aberrations are sufficiently small. All HRTEM images shown here were recorded on a 2 k × 4 k pixel CCD camera at a magnification of 1.5 M× using an acquisition time of 1.0 second and a negative C_S_^17^, under which all atoms are able to be imaged as bright dots with different bright contrasts. The sampling rate of the image is 0.008369 nm/Pixel. The images were recorded firstly along [110], and then [100] orientation in the identical position of the interface by successively tilting the specimen within a very short period in the microscope. The same GMDs could thus be imaged at two different crystallographic orientations. In addition, since Cu atoms possess a high mobility under the electron beam, Cu diffusion can occur even at room temperature^18^. Therefore, it might slightly modify the morphology when exposed to intensive electron beam for quite a long period. Precautions should be taken during the imaging of the Cu-MgO interface.

**Figure 1:**
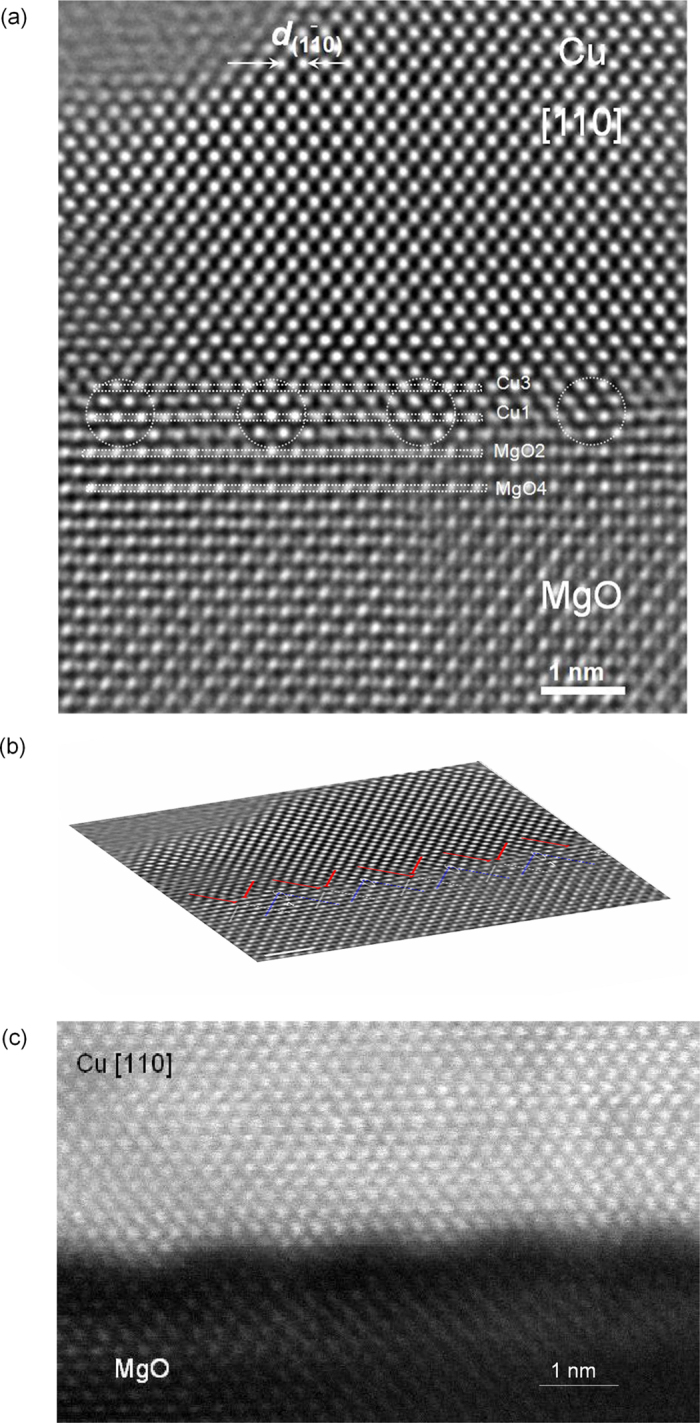
(**a**) One HRTEM image of the Cu-MgO interface with an epitaxial relationship, i.e. [100] (001) Cu // [100] (001) MgO, recorded along the [110] direction using a small negative C_S_, under which the individual O, Mg and Cu atomic columns are clearly imaged as bright dots. Between the dislocations, periodic intensity modulation mostly in the Cu side at near the interface appears which is approximately indicated by circles. (**b**) An inclined view image of (**a**), from which the dislocation components from 〈100〉 dislocation network can be explicitly identified. The {111} atomic planes in MgO crystal (pure O atomic planes) extend into the Cu, forming the incoherent areas, where GMDs (red and white lines) are located, as well as those planes (pure Mg atomic planes) into the Cu crystal, forming coherent areas (blue lines) can be easily discriminated. Taken together, Cu-O bonds are formed at the coherent regions while in betweens the Mg-Cu bonds are most likely formed. The image is Wiener filtered. (**c**) HAADF-STEM image shows the contrast difference at the coherent regions and dislocation cores (stronger dark contrast) and contrast periodic change.

Based on the C_S_-corrected HRTEM images, we can measure the Cu-Cu separations at planes parallel to the interface. To detect the influence of GMDs and probe the structural information of local atoms, the line-profiles on the first layer (Cu1), third layer (Cu3) (as indicated in Fig. 1a) were acquired. For comparison, two line- profiles in MgO (the second layer, MgO2, and the fourth layer, MgO4) were obtained as well. The atom column positions were carefully measured by fitting a Gaussian function around each maximum of a brightness line profile taken on the Cu and MgO side using a Digital Micrograph program, and then the accurate center position for each maximum as found by fitting is assigned as the center of atom column position. By this way, atom column positions can be determined at a picometer precision^19^. The Cu-Cu separations are plotted over a distance of total 17 Cu *d*_(1–10)_ separations, around 4.7 nm in length, containing three paired dislocation components (supplementary, Fig. S2).

The specimen thickness is at a range of 2.0 ~ 3.0 nm as determined by a combination of a focus series of images and image calculations. A focus series of images were recorded using a focus interval of 1.40 nm during the experiment. Image calculations were conducted using the Java EMS software^20^, a thickness focus map was firstly calculated, and then compared with the focus series of images acquired. Using this map, the specimen thickness range can be well determined.

Two clippings of the calculated images based on the standard cubic MgO (lattice constant *a*_MgO_ = 0.42105 nm) and Cu structure (lattice constant, *a*_Cu_ = 0.36148 nm) are inserted in the micrograph (supplementary, Fig. S2). These two images were calculated using Bloch wave method under the following conditions: high voltage 200 kV, defocus spread 5.3 nm, beam half convergence angle 1.6 mrad, specimen thickness of 2.0 nm, a defocus of 5.4 nm (over focus), and a negative spherical aberration (C_S_) –0.011 μm, and other aberrations (axial comma, 2-fold astigmatism) are set to be quite small which are comparable to experimental values. The simulated images confirm that the bright dots in the experimental image correspond to atom columns under the negative C_S_ imaging condition.

The atomistic structure of Cu/MgO interface is calculated by an energy minimization method theoretically with the Chen-Möbius inversion potentials, implemented by a LAMMPS program^21^. The force-field of the interface system consists of three sets of potentials for Cu slab, MgO slab and Cu/MgO interface respectively, are obtained by the Chen-Möbius inversion method for crystal^22^,^23^ and interface^24^,^25^.

The interface model has 30 monolayers at Cu slab and MgO slab respectively, and has 7 × 7 Cu cells vs. 6 × 6 MgO cells at the interface to represent a lattice misfit, where the 7:6 misfit is determined by the lattice constants of Cu and MgO crystals. Based on this model, the energy minimization calculation is implemented by a conjugate gradient method with a force convergence tolerance of 10^−8^ kcal/mol/Å per atom. To compare with experiments, a set of inter-layer distances and inter-atom distances are extracted from the optimized interface structure.

Density functional calculations (DFT) were performed with the VASP code, we used GGA-PBE for exchange correlation and projector augmented wave (PAW) potentials. The cutoff energy for the plane-wave basis was 400 eV. Gaussian smearing was used for the orbital occupancies. The calculated lattice constants are 3.64 Å for fcc Cu and 4.24 Å for bulk MgO, respectively. 2) The misfit between Cu (001) and MgO (001) lattice is about 14%. For the coherent interface structure, we expanded the in-plane lattice constant of Cu to fit the MgO part, because Cu is softer compare with MgO. A 21 × 21 × 1 mesh was used for the k-point sampling in the Brillouin zone (BZ). Geometry optimizations of atomic positions as well as the cell parameters were performed to obtain the optimized interface structure.

## Results and discussion

### Atomic structure of GMD from [110]

Figure 1a shows a C_S_-corrected HRTEM image along [110] direction, where the individual Cu, Mg and O atom columns are clearly imaged as bright dots with a relatively weak intensity for O columns. The interfacial structures can be readily identified, where the first MgO layer (assigned as interface layer) is somewhat disturbed compared with the first Cu layer. Along the [110] orientation, the paired GMDs consist of two components projected from the 〈100〉 direction dislocation network (which is easily seen in the following schematic drawing). An inclined view of the image simplifies to visualize one component of the projected Burgers vector (Fig. 1b). Looking along the {111} planes, oxygen atom planes in MgO (white lines, weak bright contrast) extend into the Cu crystal (*red lines)*, forming an incoherent area, where the dislocations are located, whereas between the dislocations Mg atom planes (*blue lines*, strong bright contrast) extend into the Cu crystal, forming a coherent area. Coherent areas are more obvious in Fig. 1a, and approximately labeled by white circles, where Cu sits on top of O, i.e. Cu-O bonds must have formed. Between the coherent areas, the GMD cores are located, where Cu atom sits on top of Mg atom, i.e. Cu-Mg bonds must have formed (supplementary, Fig. S2). From HAADF–STEM image (Fig. 1c), the GMD core between coherent regions appears as a more dark contrast, which is due to the large atomic potential around the dislocation cores. Several GMD cores at the interfaces are clearly visible reflecting a periodic change in contrast. The bond lengths of Cu-Mg are larger than those of Cu-O at the coherent region as revealed by atomic model analysis and density functional theory (DFT) calculations^11^.

The obtained HRTEM image further enables us for quantitative analysis of the bond length. The bond length of Cu-O at the coherent region (O-atop) can be directly determined from the image. By using the intensity profile and followed by fitting analysis (supplementary, Fig. S3), a measured value of (0.216 ± 0.008) nm is acquired, which is in good agreement with the calculated by DFT, e.g. 0.216 nm as reported by Matsunaka *et al.*^11^.

Based on the C_S_-corrected HRTEM image, the atomic structure of the dislocation core can be well distinguished, which was hardly possible using conventional HRTEM imaging in the past decades^1^,^8^. Three paired dislocations are identified (supplementary, Fig. S3), the terminated plane position, however, differs appreciably in certain dislocation cores. An enlarged image of one paired dislocation core and the corresponding atomic model (Fig. S4) clearly reveal the Cu-O bonds formed at the coherent regions and the atomic configurations of dislocation core in betweens. Overall, the atom configurations of GMD cores slightly vary from one to another. A further analysis reveals that the GMD might terminate at slight different position with one atomic layer shift upward or downward relative to the interface layer.

### Atomic structure of GMD from [100]

**Figure 2:**
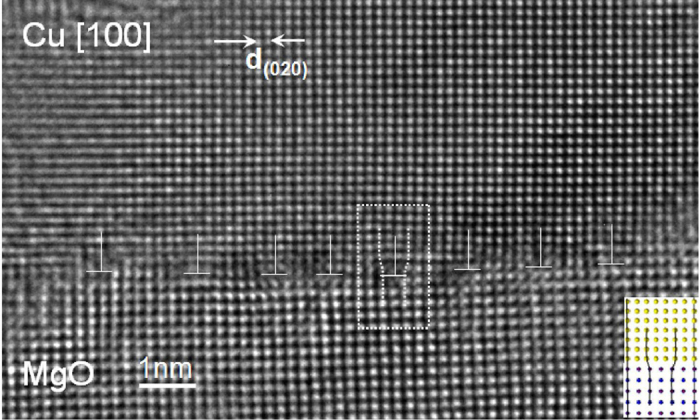
HRTEM image taken along the [100] zone axis from the identical interface position as in Fig. 1a tilting by 45° with respect to Fig. 1a. In this projection, Mg and O atom columns overlap and cannot be discriminated. The misfit dislocations with full Burgers vector are visible. 8 GMDs are contained in this micrograph. Compared to Fig. 1a, the distance between the GMDs becomes smaller due to the image projection.

**Figure 3:**
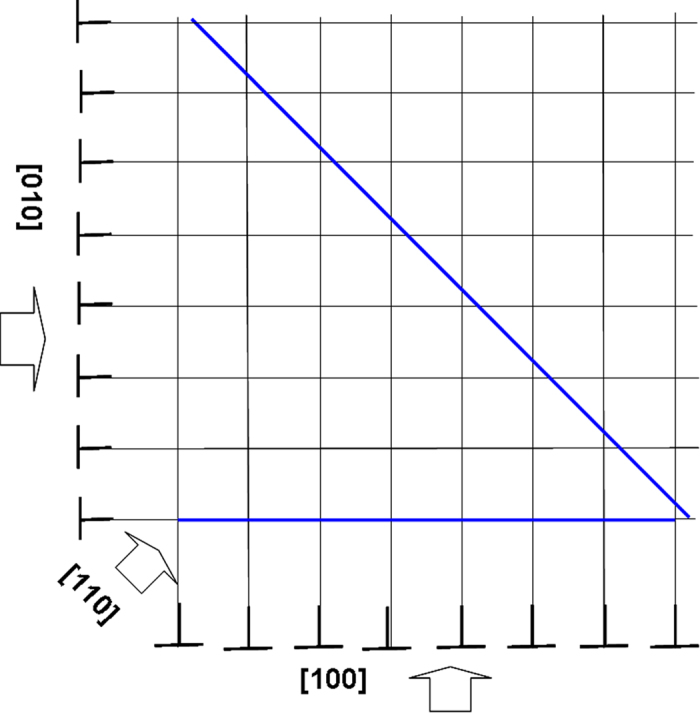
Schematic drawing of the edge-type GMD network at the interface plane with [100] and [010] line directions and Burgers vector *b* = 1/2*a*〈100〉, i.e. a is the lattice parameter. Imaging along [100] reveals one set of the GMD edge-on while in [110] zone axis a projection of both sets is imaged.

The HRTEM image obtained from the [100] direction resolves the interface GMD core edge-on (Fig. 2) in contrast to the [110] HRTEM image. It was acquired from the same interface position as Fig. 1 and by successively tilting the specimen by 45° in the microscope. An array of GMDs was observed with a certain distance. The schematic drawing (Fig. 3) shows a dislocation network laying at the interface plane consisting of two perpendicular sets of 〈100〉 edge-type dislocations. In [110] zone axis only components from both sets of dislocations and their Burgers vectors are projected in the image. Because of this projection, the GMD spacing appears wider by a factor of √2 in the [110] direction compared to the [100] direction (Fig. 3) where the full Burgers vector is resolved. In total, eight GMDs are contained in the micrograph (Fig. 2). The first 5 dislocations (from right to left in sequence) in [100] projection correspond to 5 left-inclined dislocations viewed along [110] direction (from right to left, Fig. 1b). The dislocation cores are more apparent than those in [110] direction, however, with slightly blurred contrast on some cores due to the strong strain existing. A corresponding atomic structure for a representative dislocation core (denoted by an area) is inserted in the corner. The experimental measured distance between the GMDs is (1.24 ± 0.18) nm, which agrees quite well with the theoretically predicted, i.e. 1.28 nm by using 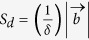, where 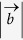 is the magnitude of the Burgers vector of the dislocation, i.e. 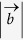 = 0.18074 nm, and δ ≈ 14.1% is the mismatch of the two lattices.

## Quantitative atom analysis

### Experimental measurements

**Figure 4:**
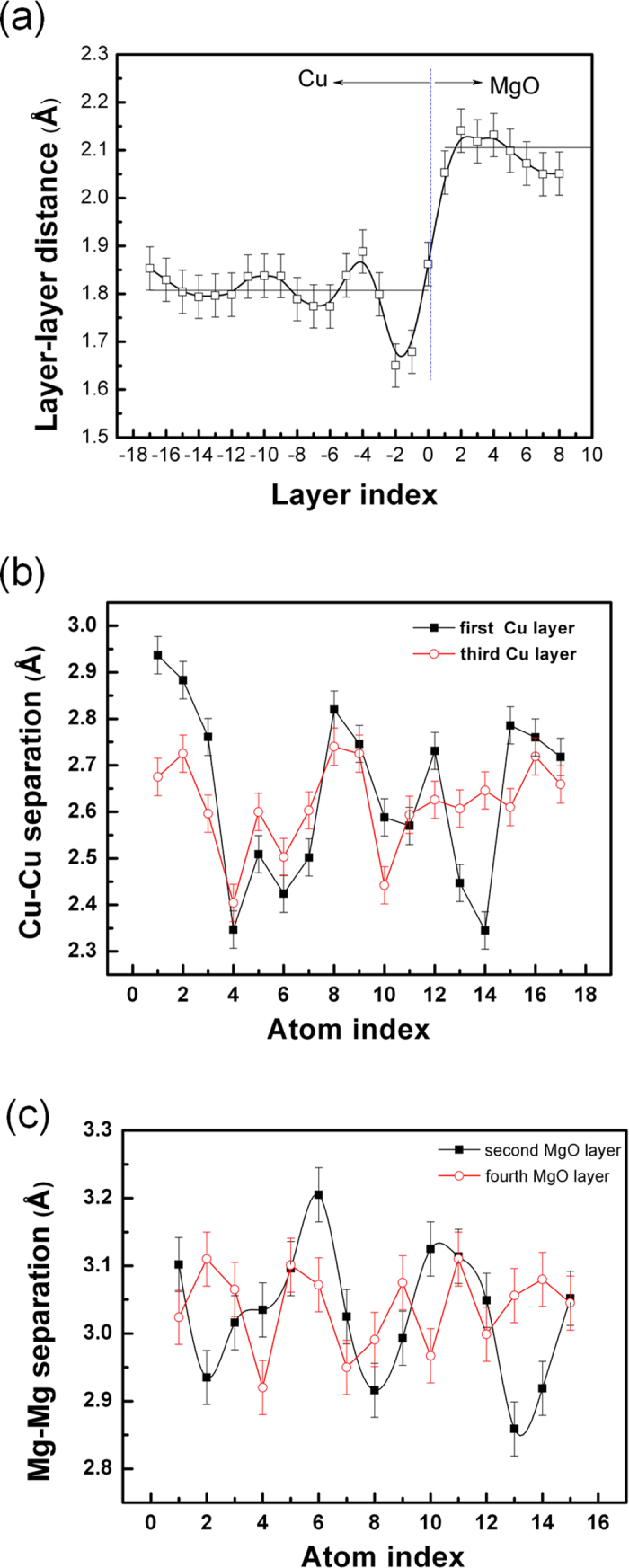
The experimental measurements on atomic position, (**a**) the variations of the interplanar spacing (*d*_*002*_) across the interface plane is plotted as a function of interplanar spacing numbers in Cu and MgO. The thin lines denote the bulk Cu *d*_*002*_ and MgO *d*_*002*_ spacing, respectively. The interface location is indicated, representing the spacing between the first Cu layer and the first MgO layer). Panel (**b**) shows the variations of Cu-Cu separation in the second layer (Cu1), the third layer (Cu3) over a distance of several nanometers along the Cu planes parallel to interface (labeled in Fig. 1a) starting from left to right. In total, 17 Cu-Cu separations are included. Larger fluctuation amplitude in the first several Cu-Cu separations may result from subtle local contrast variations. Panel (**c**) shows the variations of Mg-Mg separation obtained from the second MgO (MgO2) and fourth MgO layer (MgO4).

To examine the subtle displacement of atom positions at or near the interface in a more precise way, atomic column quantitative analysis were applied. Here, the variations of interplanar spacing (layer–layer distance, or L-L distance) across the interface were explored. The (002) plane spacing, *d*_*(002)*_, parallel to the interface, is carefully determined and plotted as a function of the distance over several nanometers across the interface. The measured results are presented in Fig. 4a, where, in total, 17 *d*_(002)_ in Cu and 10 *d*_(002)_ spacings in MgO are included. Please note that the error bar are due to: (1) multiple measurements; (2) fitting process.

The atomic displacement distribution obtained from the experimental image reveals an obvious change in the interplanar spacing when crossing the interface. A maximum in the *d*_(002)_ is approached from the MgO side at the first few layers near the interface, whereas the spacing dramatically decreases at the Cu side, then followed by the seemingly oscillations till it almost approaches a invariable value. The variations of the interplanar Cu (002) spacing adjacent to the interface exhibits obviously oscillatory behavior relative to the bulk Cu (002) spacing and with a wavelength of 6 ~ 7 *d*_(002)_ spacings. These oscillations do not follow the periodicity of the lattice. In contrast, the oscillations at MgO are less pronounced. This is the first experimental observation regarding the damped oscillatory behavior of the interplanar spacing at interfaces. The oscillations are probably relevant to the strain fields from the GMDs and uncompensated mismatch strain in the coherent interface region. Seemingly, the phenomenon shows a similarity to the *Friedel* oscillation present at metal surfaces^26^,^27^,^28^. The physics origin of the oscillatory interplanar spacing, i.e. contraction and expansion in *d*-spacing, might be attributed to the repulsive or attractive atomic forces between the layers induced by electronic charge redistributions.

To further probe the structural information of the local atoms at or near the Cu-MgO interface, we measured the Cu-Cu and Mg-Mg atom separations at planes parallel to the interface at a picometer precision. Fig. 4b is a plot of the resulting separation between subsequent Cu atom columns as a function of distance along the interface, about 4.7 m in length and covering the two pairs of dislocation cores, for the first layer (Cu1) and third layer (Cu3) (labelled in Fig. 1a) with respect to the interface. It shows that nearly periodic fluctuations of separation take place very pronouncedly in the Cu1 (the maximum difference is set to the maximum Cu-Cu subtracts the minimum Cu-Cu separation, ∆ ≈ 0.6 Å). Near to the dislocation core, Cu-Cu separation becomes smaller. The magnitude of Cu-Cu separation in Cu3 is relatively reduced. The periodic fluctuations in the Cu3 become less significant. The remarkable change in the Cu-Cu separation is essentially attributed to the GMDs.

Similar measurements performed on the MgO side show that the variations of Mg-Mg separations are much less pronounced compared to the Cu-Cu due to stiff bonds reflected in a dramatically higher Young’s modulus (Fig. 4c). Mg-Mg separation in the second layer (MgO2) shows periodic variations (as the first MgO layer is somehow distorted, we start measuring from MgO2, the maximum difference, ∆ ≈ 0.35 Å), and the maximum is reached at the dislocation core positions. At the fourth layer (MgO4), the magnitude of separation is relatively reduced. This for the first time experimentally directly reveals the nearly periodic fluctuation behavior of the local atoms adjacent to the interface and also illustrates to what extent the GMD affects the local atomic arrangements at neighboring atomic layers near the interface. It is anticipated that the atom nearly periodic fluctuation behavior will be useful for understanding the fundamental role of interfaces in materials at the atomic scale.

### Theoretical calculations

**Figure 5:**
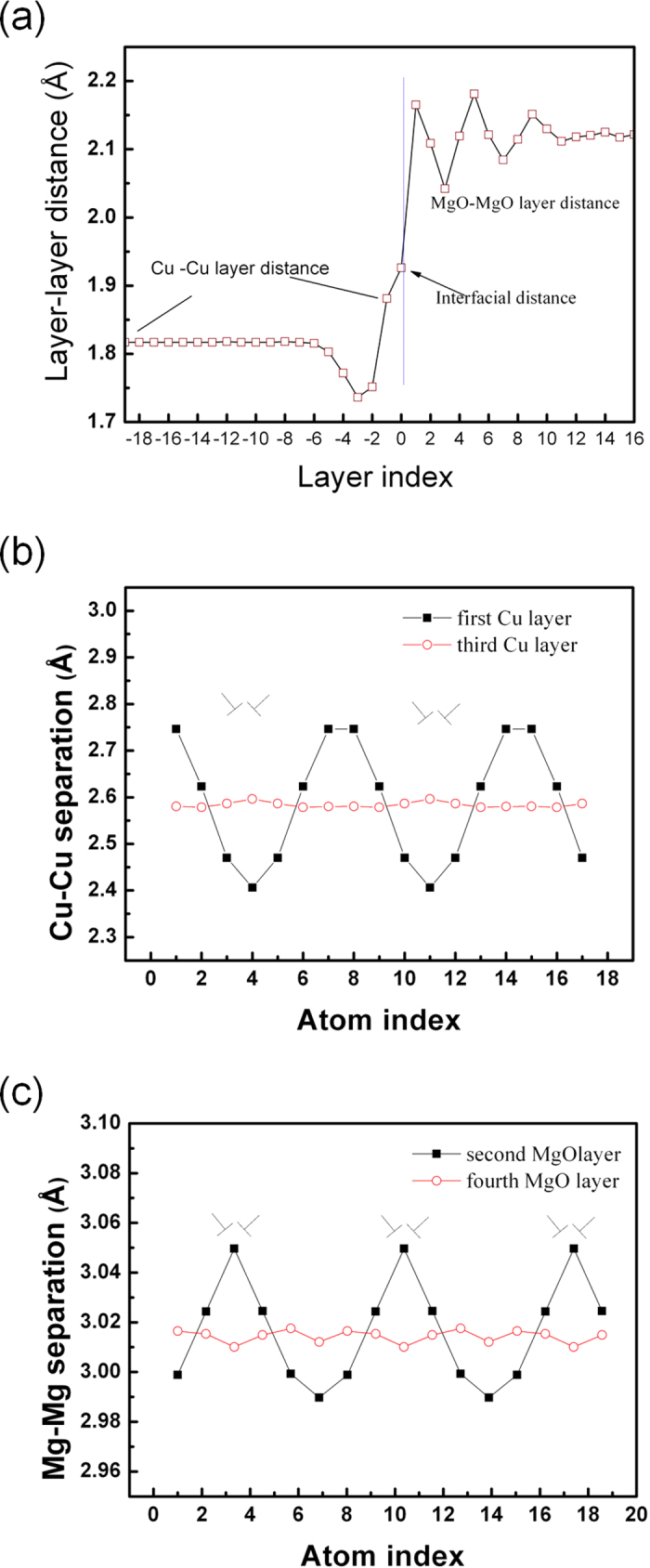
The theoretical calculation about atomic position, (**a**) the variation of the layer –layer (*d*_*002*_) distance across the interface plane is plotted; Panel (**b**) shows the variations of calculated Cu-Cu separation in the first layer and third layer; (**c**) shows the changes of calculated Mg-Mg separation in the second and fourth layer.

The calculated results using the conjugate gradient methods are shown in Fig. 5. The distance of layer-layer parallel to interface is plotted as a function of layer index (Fig. 5a). To a certain degree, it exhibits a similarity to the experimental result (Fig. 4a), particularly the layer-layer distance in the first several Cu planes are obviously decreased, but with a less magnitude relative to experimental results. After about 5 layers in Cu the layer distance approaches to a constant, instead an oscillation in Cu. Surprisingly, the layer-layer distances calculated in MgO somewhat oscillates adjacent to the interface, which is not very pronounced in the experiments.

Calculated Cu-Cu and Mg-Mg separations at the first and third layer are also shown in Fig. 5b,c. Due to the effect of dislocation core, Cu–Cu and Mg-Mg separations at the first layer are strongly oscillated, which is also reflected in the experimental plot. Relatively, the magnitude of calculated oscillation is smaller (the maximum difference for Cu-Cu separation, ∆ ≈ 0.4 Å; for Mg-Mg, ∆ ≈ 0.06 Å). The interfacial GMD periodically modified the atomic spacing. However, this modification becomes weaker or completely disappears at the third layer in contrast to such experimental results. The experimental observation indicates the effect of the interfacial GMD still exist up to the third and fourth atomic layer while the magnitude of atomic separation changes is obviously reduced relative to the first Cu and MgO layer. At the dislocation core, Cu-Cu separation shows maxima while Mg-Mg separation nearly becomes minima as seen in Fig. 5b,c. Both display a periodic change along the interface plane.

## Strain measurements at the dislocation

**Figure 6:**
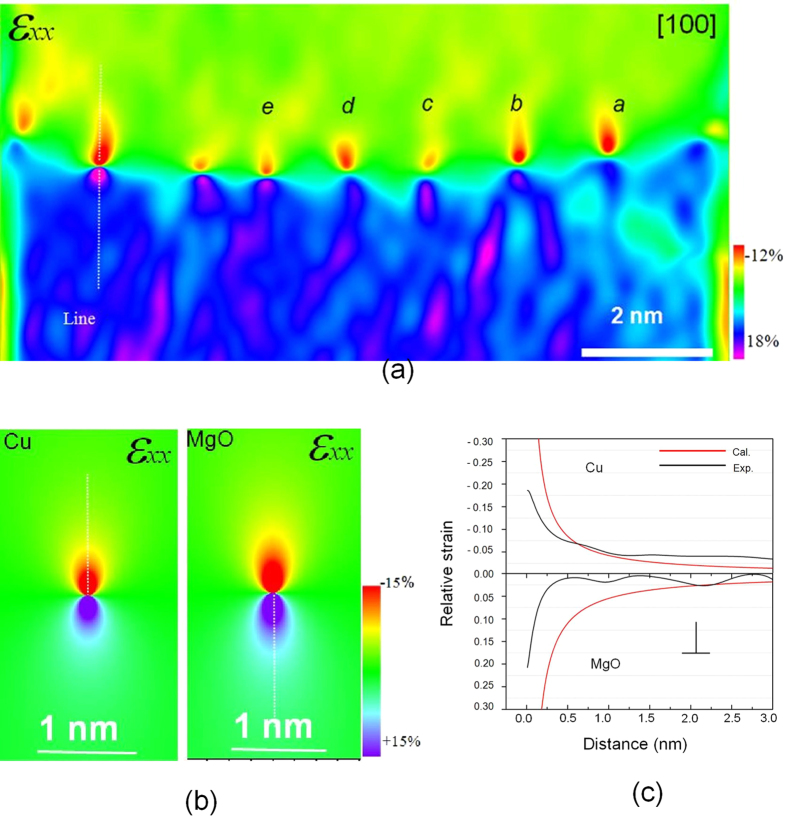
The strain field distribution around the GMD core, (**a**) strain field color map obtained based on the [100] HRTEM image (Fig. 2). (**b**) The calculated strain map (*ε*_*xx*_) of edge dislocations in Cu and MgO using the classic Peierls-Nabarro model. (**c**) A quantitative comparison of strain, *ε*_*xx*_, plotted as a function of distance away from the dislocation core, is made between the profiles (dark) acquired along the line in (**a**) and the profiles (red curve) along the lines in the calculated Cu and MgO maps in (**b**). In Cu, *ε*_*xx*_ is negative, representing a compressive strain; while in MgO *ε*_*xx*_ is positive, representing a tensile strain. The calculated curves in (**c**) show a reasonable match for both Cu and MgO at some distances from the dislocation core.

According to the C_S_-corrected HRTEM images (Fig. 1a and Fig. 2), the strain fields around the GMDs are approximately mapped using the geometrical phase analysis (GPA) technique^29^,^30^. The strain component *ε*_*xx*_, obtained from the [100] projection (shown in Fig. 6a), denotes the strain distribution parallel to the interface. The *ε*_*xx*_ map clearly shows that adjacent to the core the Cu lattice is subjected to compression with respect to the MgO lattice. Moreover, the MgO lattice around the core is expanded as compared to the bulk MgO. Fig. 5a also displays that the relative magnitude of strain varies slightly at dislocation core.

To approximately evaluate the strain distribution, the theoretical strain field from a single misfit edge dislocation was calculated because the theoretical model is more complicated if the interaction between GMDs at the interface is considered, so far, no analytical solution model is available for an array of interacting dislocations. The displacement of an edge dislocation can be therefore expressed by isotropic elastic theory or the classic continuum Peierls-Nabarro model^31^ (supplementary materials Fig. S5), along the x direction, which is the direction parallel to the interface plane, as: *u*_*x*_, and the plane strain is described as the follows: 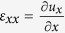, where *b* = 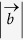 is the magnitude of the Burgers vector of the edge dislocation, i.e. *b* = 0.18074 nm for Cu, and 0.21053 nm for MgO.

For simplification, both Cu and MgO are here treated as isotropic materials (a Poisson’s ratio ν_Cu_ = 0.36 and ν_MgO_ = 0.18) for calculating the individual strain fields around an edge dislocation. The calculated strain field distributions of an edge dislocation in Cu and MgO are nearly the same (see Fig. 6b), and both exhibit a considerable similarity with the experimental map (Fig. 6a). To further visualize the subtle difference, line profiles across the dislocation strain field (as labeled in Fig. 6a,b) in Cu and MgO are acquired. Here, the calculated strain field in Cu (the upper part) and MgO (the lower part) are taken for comparison with experimental strain field. Two line profiles drawn in the experimental map across Cu into MgO at two dislocations, together with the calculated profiles (red), are plotted in Fig. 6c, where Cu shows a compressive (negative) strain while the MgO possess a tensile (positive) strain relative to bulk MgO. To a certain extent, it reveals the discrepancy between the calculated and experimentally measured strain fields. Apparently, at the core a large deviation exists whereas with increasing distance from the dislocation core a better agreement is reached for both the Cu and MgO side.

From atomic quantitative analysis, the oscillations of L-L distance in Cu adjacent to interface are experimentally observed. The observations are partially reproduced by theoretical calculations. In Cu side, the calculations predict the almost same behavior for the first several layers. It means that the L-L distance of the first several Cu layers is remarkably reduced, which could be due to relative soft reflected in a dramatically lower Young’s modulus (110 ~ 128 GPa) as compared to MgO (270 ~ 330 GPa). However, the oscillation is not well repeated theoretically. Instead, adjacent to the interface, L-L distance in MgO shows a small oscillation in the first few layers. The reason for such difference between calculations and experiments is still unclear. Perhaps, a ‘good’ interatomic potential model might be still needed for metal –ceramic interface. Although this, in contrast to the calculations using DFT or phenomenological methods using va very limited atom numbers^11^,^32^, the optimized process using conjugate gradient method which can handle a large supercell may be suitable for such complex metal-ceramic interface.

DFT calculations using coherent interface structure with a simple model were also carried out in this study on a supercell (13 layers of Cu and 13 layers of MgO to construct the coherent interface), the result for the L-L distance only shows a marginal oscillation in Cu (with a very small magnitude) while hardly change in MgO side. There is a large discrepancy between the calculated and the experimental results, particularly the oscillation magnitude and the periodicity. Obviously, such simple model does not allow us to capture the complex geometric features in a real interface structure. The large interface strain in the coherent interface model is one of the main reasons. Therefore, a more reasonable way to solve this problem is to use the incoherent interface model. This requires to accommodate the in-plane lattice parameters by matching 7 × 7 Cu layers with 6 × 6 MgO layers, The system size is too large to handle by DFT calculations. From this viewpoint, empirical method described in the above is more applicable. On the other hand, it also implies that other factors underlying which are not considered in the calculations may play a role in the Cu relaxation at the interface. It is still not well understood. In short, the theoretical calculations by empirical method can partially reproduce the Cu relaxations (for the first few atomic layers), however, not completely.

It is seen that Cu-Cu and Mg-Mg separation also exhibits slight deviation between the theoretical calculation and experimental observations. The difference may be understood from the atom disorders at the first interface Cu and MgO layer, interface intermixing within one atomic layer may occur, and the interface vacancies may also be present, all of which are not taken into account in the calculations. These defects could somehow change the atomic column positions, and to a certain extent, influence the results.

The existing discrepancy in quantitative strain evaluation may be attributed to the following factors: (i) the assumption made in the calculations, i.e. isotropic elastic behavior. Actually, neither Cu and MgO nor the interface is isotropic. (ii) In the case of the interface, the strain tensor measured by GPA corresponds to the relative distortions of one lattice with respect to the reference lattice, which is in reality different from the usual strain tensor. This could give a slight deviation between the calculated and the experimental map. (iii) The interaction between geometrical misfit dislocations at the interface is not included. The dislocation interaction obviously affects the strain fields especially when the dislocation distance is in a range of a few nanometer ranges. This is not considered by a simple theoretical model.

**Figure 7:**
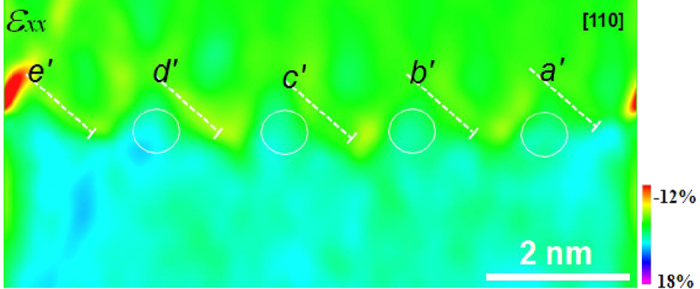
Strain field map, *ε*_*xx*_ , obtained from the [110] HRTEM image (Fig. 1a), revealing the non-identical strain distribution from both sets of 〈100〉 GMDs as labeled.

The strain map, *ε*_*xx*_, shown in Fig. 7 (*ε*_*xy*_, *ε*_*yy*_ and rotation maps shown in Fig. S6) from [110] shows a relatively small amount of strain distribution around the core as compared with the [100] strain map (a magnified color scale is applied to visualize the small strain). Left-inclined dislocation components in the [110] map (as indicated in Fig. 7) are actually the projection of corresponding dislocations in [100] direction (those right-inclined dislocations are projected dislocations along [010] direction). Therefore, the strain field map in [110] originates from a summation of a certain percentage of strain field projected from the [100] map and a strain distribution perpendicular to the dislocation line, i.e [010] map (which was not observed in this experiment). This denotes that the strain distribution of a GMD at the Cu-MgO interface can be visualized by successively imaging along two crystallographic orientations, i.e. [100] and [110]. It also means that the strain components of GMDs perpendicular to those GMDs being viewed are possible to be detected via imaging method in a microscope with a large *pole-piece* gap. Moreover, close examination of Fig. 7, it is found that the strain distributions from two sets of 〈100〉 GMDs are not identical due to several reasons, such as the different line length in the cross-sectional sample, electron beam effects etc. A nearly 3-D reconstruction of the strain field around an edge dislocation is likely to be made when a series of images acquired.

## Conclusions

In conclusion, we have shown the atomic structures of dislocation core and the peculiar behavior of atoms at and near a Cu-MgO interface with the use of quantitative measurement of atom column positions and theoretical calculations, such as variations of interplanar spacings and periodic fluctuations in atom separation induced by GMDs. At the coherent region, the atomic bond length of Cu-O is directly measured with a picometer precision. Successively imaging on the dislocation core along [100] and [110] direction made it possible to visualize the strain fields of dislocation at a nearly 3D view, which reveals that the GMDs show subtle difference in their strain fields due to the minor difference of corresponding atomic structure.

The phenomena were not possible to be well detected in the past decades because of the inaccessibility of the high-precision measurements primarily ascribed to the limitation of instruments. Practically, very local atomic structural variations at or near interfaces can lead to a degradation of electronic devices in use, and thus limit the performance and lifetime. For nanostructured materials, the oscillations may be limiting the resulting hardness as recently reported by theoretical calculations^33^. Thus, studies dealing with the fundamental and practical aspects of heterointerfaces between materials is of great importance for further understanding the properties of interface-controlled materials in general; in particular, for their future applications such as in microelectronic devices or for tribological purposes.

## Additional Information

**How to cite this article**: Zhang, Z. *et al.* The peculiarity of the metal-ceramic interface. *Sci. Rep.* **5**, 11460; doi: 10.1038/srep11460 (2015).

## Supplementary Material

Supplementary Information
