# Supplementary material for: The peculiarity of the metal-ceramic interface
Source: Sci Rep. 2015 Jun 19;5:11460. doi: 10.1038/srep11460 (PMC4473597; doi:10.1038/srep11460)

Supplementary Materials for

"The peculiarity of the metal-ceramic interface"

Zaoli Zhang[1, *], Yao Long[2], S. Cazottes[1,#], R. Daniel[3] , C. Mitterer[3], G. Dehm[4]

*To whom correspondence should be addressed. E-mail: zaoli.zhang@oeaw.ac.at

Text for Figs. S1-S6

Fig.S1

Fig.S1 is a drawing (top view) of the sample geometry, which schematically illustrates the sample cutting direction and the dislocation network lying at the interface. The cutting was aligned at an angle of 22.5° relative to the <110> and <100> directions, which enables to reach two crystallographic orientations by tilting a large angle in our microscope. The blue lines are the projected planes, which demonstrate the dislocation configurations at the interface when viewed along <110> and <100>, respectively.

Fig.S2

The O-lattice theory predicts that the interfacial atoms are located at their most energetically favorable position, creating a square array of good match points (O-points), separated by misfit dislocations. Interfacial atomic structures using O-lattice theory model are shown in Fig.S1, viewed along the different orientations, such as side view [110] and tope view [001]. Along the [110] orientation, two-paired dislocations are included, and three Cu-O bonds at the good match (coherent) regions are indicated in an elliptical circle. As a guide, the corresponding {111} atom planes running through at the good match regions and dislocations are labeled by lines at two viewing directions. In the top view, O-points are clearly seen.

Fig.S3

(a) The specimen thickness is at a range of 2.0 nm to 3.0 nm as determined by a combination of focus series of images and image calculation. The paired dislocation components projected from the <100> direction network are visible, and separated by 'good match'--coherent regions giving a little bit strong brighter contrast (approximately indicated by circles in Fig.1a). At this position, Cu sits on top of O, i.e. Cu-O bonds are formed. Three paired dislocation components are covered in this micrograph.

(b) An inclined view image, in which one dislocation component from <100> dislocation network can be explicitly identified. Looking along the {111} planes, oxygen atom planes in MgO (white lines, weak bright contrast) run through the Cu crystal *(red lines*), where the dislocations are located, whereas between the dislocations Mg atom planes (*blue lines,* strong bright contrast) extend into the Cu crystal, forming a "good" match area. These areas are more obvious in Fig.1a, and approximately labeled by white circles. Fig.S3(a) is also used for the determination of Cu-O bond length at the coherent regions. Using bulk MgO (lattice constant 0.42105 nm) as an internal standard to calibrate the HRTEM image, the quantitative analysis is conducted on the image. Four paired dislocations (red lines) are contained in the image. Viewing along the {111} planes at an inclined angle, one Mg {111} atom plane (denoted by blue dotted lines) extends into Cu lattice, forming a coherent area, O-atop. Therefore, four coherent areas exist in the image, where Cu-O bonds are formed, labeled as number 1, 2, 3 and 4. We determine the Cu-O bond length at the coherent areas by using the intensity profile and fitted by a Split Gaussian functions. The determined Cu-O bond lengths are listed below: **No.1**, $d_{Cu\text{-}O}$= 0.2132 nm; **No.2**, $d_{Cu\text{-}O}$ = 0.2228 nm; **No.3**, $d_{Cu\text{-}O}$ = 0.2249 nm, and **No.4**, $d_{Cu\text{-}O}$ = 0.2061 nm. As shown, an averaged measured Cu-O bond length ($d_{Cu\text{-}O}$) is (0.216 ± 0.008) nm. According to the recent theoretical calculation reported by Matsunaka [1] using the DFT, the Cu-O bond length at the coherent regions is 0.216 nm, revealing a close correspondence to our experimentally measured value.

Fig.S4

An enlarged image of one paired dislocation and the corresponding atom configurations, where the coherent regions are labeled by elliptical circles and the corresponding guide lines are highlighted both in the image and model.

Fig.S5

The calculated strain maps using two different models. The **Left** map is calculated using isotropic elastic theory. The isotropic elastic theory describes the displacement of an individual edge dislocation as [2]:

$$u_x = \frac{b}{2\pi}\left[\tan^{-1}\frac{y}{x} + \frac{xy}{2(1-\nu)(x^2+y^2)}\right]$$
$$u_y = -\frac{b}{2\pi}\left[\frac{1-2\nu}{4(1-\nu)}\ln(x^2+y^2) + \frac{x^2-y^2}{4(1-\nu)(x^2+y^2)}\right]$$

Where x and y are the right –angle coordinates, respectively. b is the Burges vector and ν the Poisson ratio .

The **Right** map is calculated by using Peierls-Nabarro model. Within the classic continuum Peierls-Nabarro model, the displacement field can be descripted as (X direction *is* parallel to the interface plane) [2]:

$u_x = -\frac{b}{2\pi}\arctan\left(\frac{2(1-\nu)x}{y}\right)$, and the plane strain is described as the follows: $\varepsilon_{xx} = \frac{\partial u_x}{\partial x}$

From the calculated strain map, it is clearly seen that the classic Peierls-Nabarro model well fits to the experimental strain distributions around the geometrical misfit dislocations.

Fig.S6

The strain distribution maps around the dislocations obtained using GPA show ***Eyy***, ***Exy*** and rotation map along the [110] directions. Note that the difference at the interface.

Reference

[1] D. Matsunaka, Y. Shibutani, Phys. Rev. B 77 (2008) 165435

[2] J. P. Hirth, J. Lothe. Theory of dislocation, New York: Wiley; 1982

Fig.S1

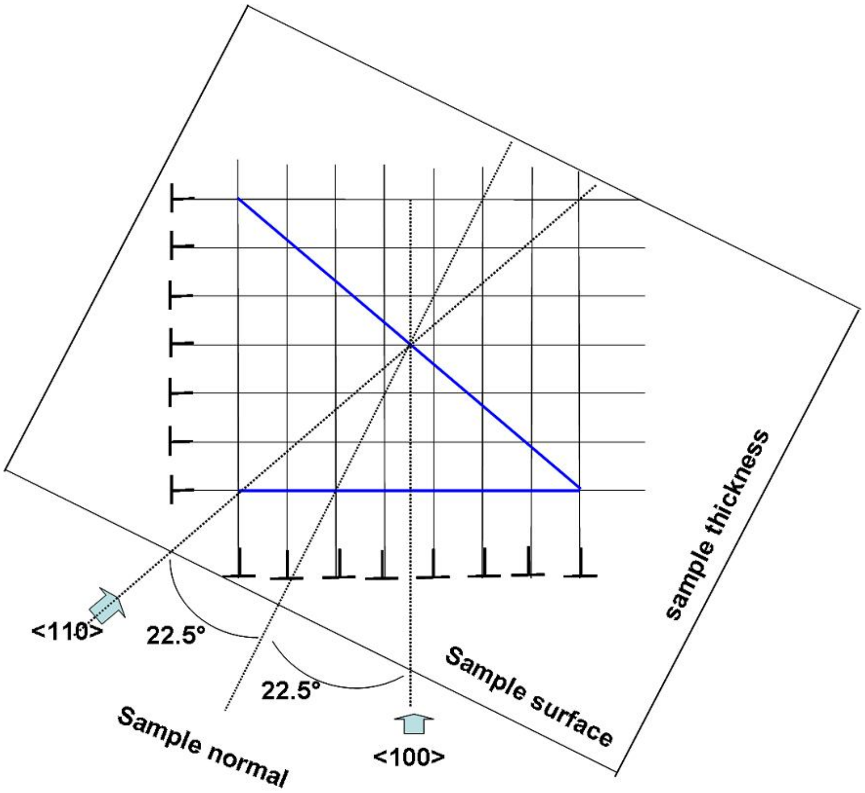

sample thickness
Sample surface
Sample normal
<110>
<100>
22.5°
22.5°

Fig. S2

Side view [110] Top view [001]

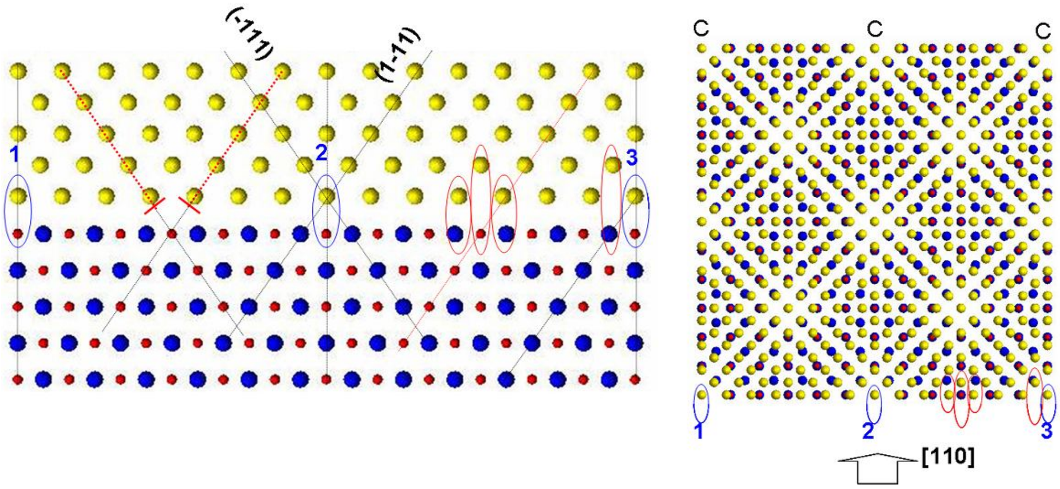

Fig.S3 (a)

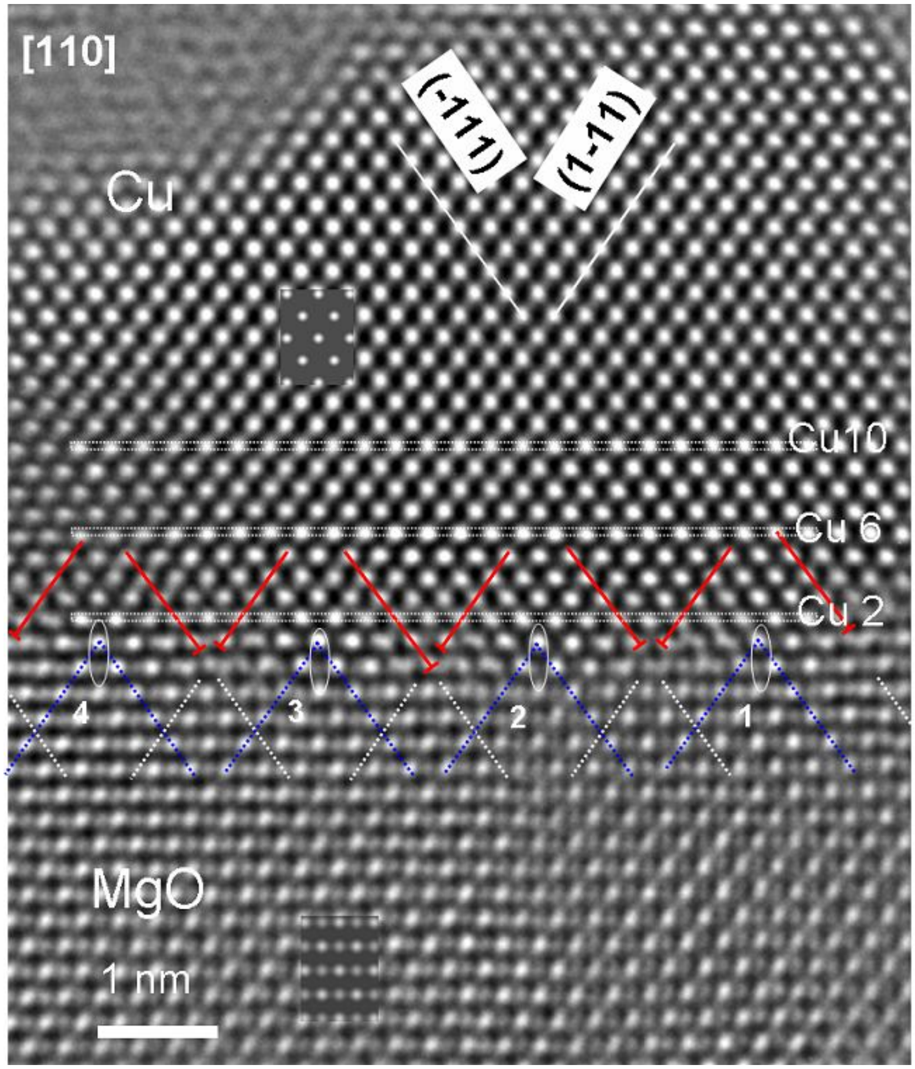

Fig.S3 (b)

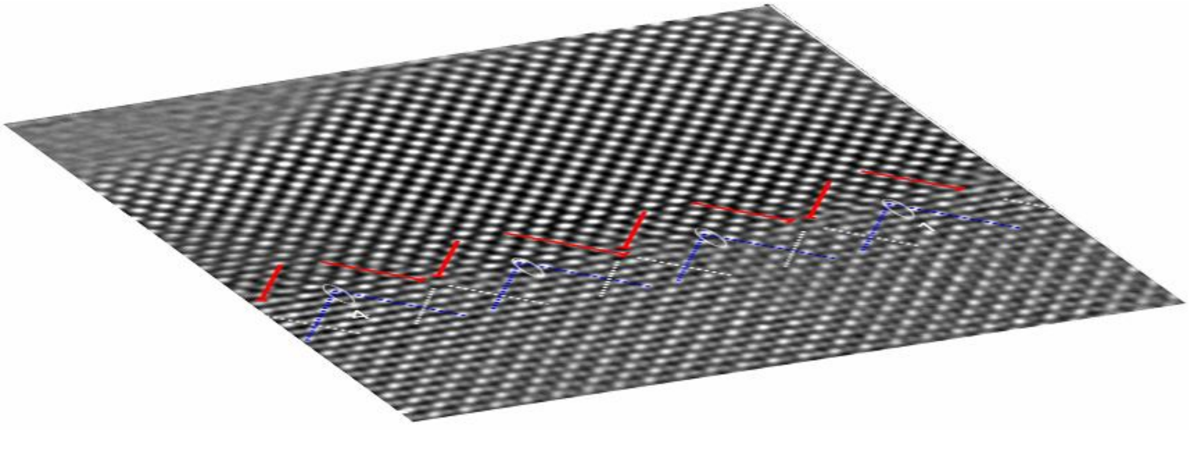

Fig.S4

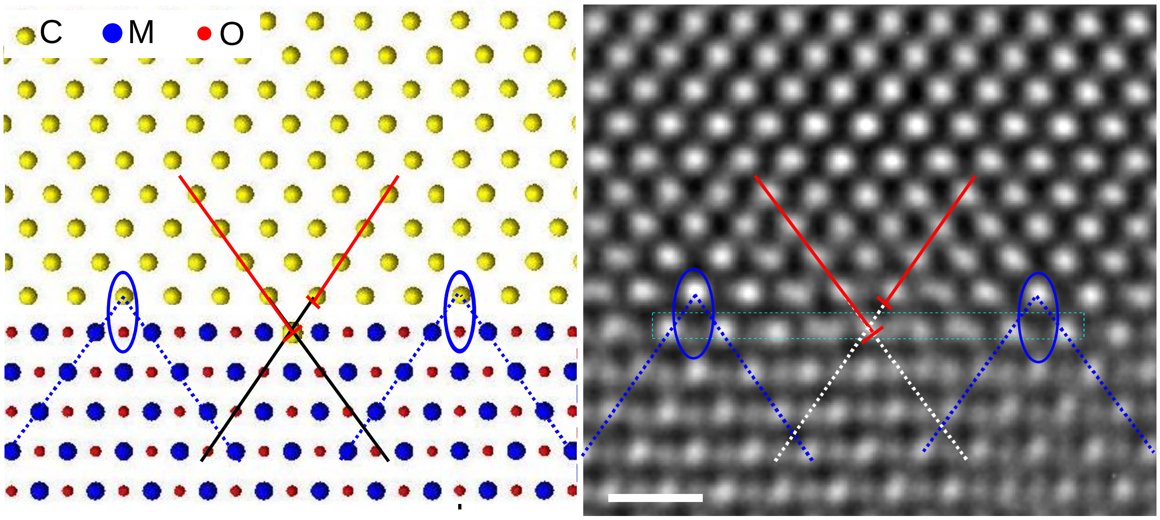

Fig.S5

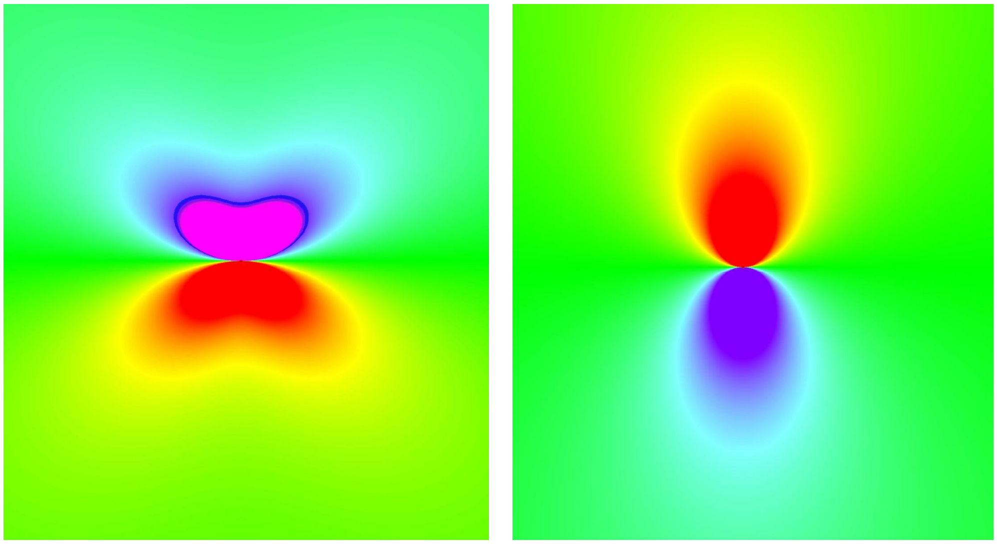

Fig.S6

Eyy

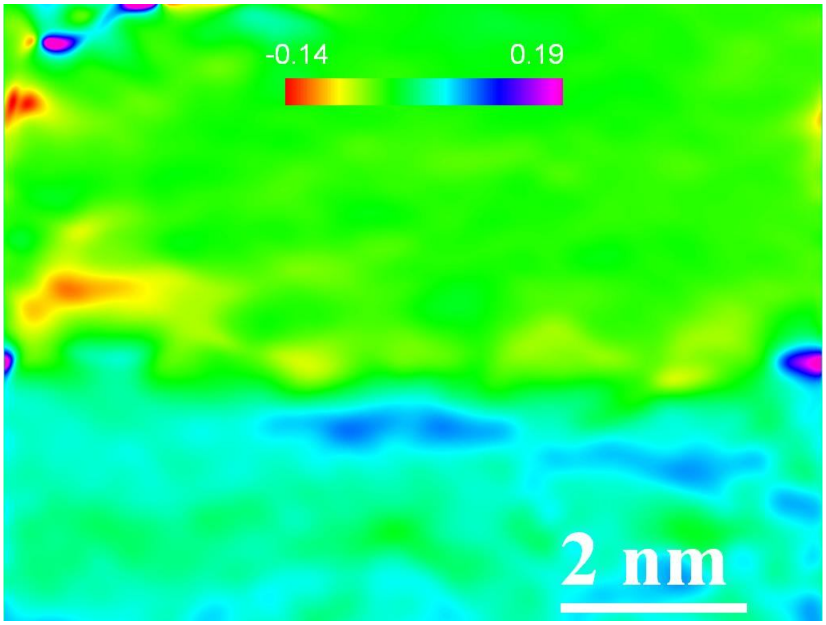

Exy

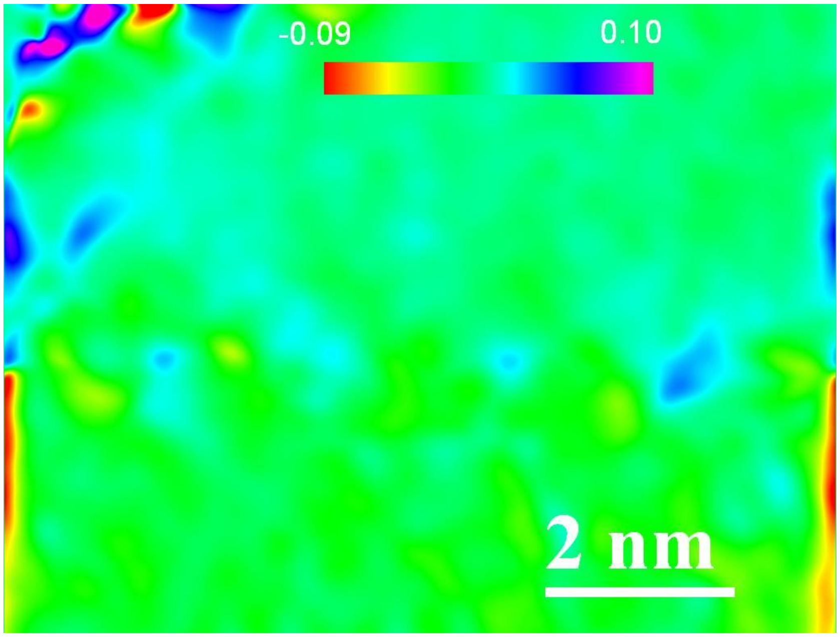

Rotation map

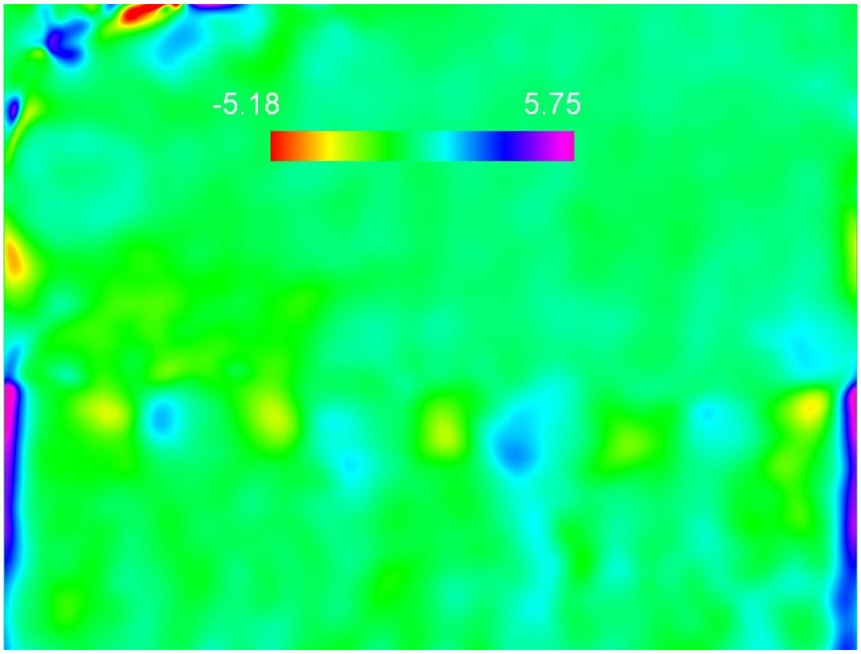

Supplement: Supplementary Information [file srep11460-s1.pdf]
